# Our Topical Index

**Published:** 1889-01

**Authors:** 


### OUR TOPICAL INDEX.

We take considerable pride in calling attention to the exhaustive cross index for 1888 found in this number. It is run as a single form and placed at the end of the reading pages, which are also full forms. It can, therefore, be taken out by cutting the wire binding-thread, and bound with volume IX. There has been considerable call for such an index, but the immense amount of labor and time necessary to compile it have no doubt kept other journals from adopting it. THE INTERNATIONAL does not intend to spare labor or money to give its readers the best journal published in this country. Send us in your subscription for 1889, and see if we do not keep our word. It will only cost you *two* dollars to find out. We do not mean by that, that we are going to reduce the price of the journal for the entire year; oh, no; but we will send a full receipt for 1889 to all who send in the above amount within the next *thirty days.* Please tell some of your brother practitioners. Don't put it off, or it will be too late for them to avail themselves of the reduction; also, don't forget that *you* can *renew* on the same terms.

